# Associations between quality of health care and clinical outcomes in patients with rheumatic and musculoskeletal diseases: a rehabilitation cohort study

**DOI:** 10.1186/s12891-022-05271-3

**Published:** 2022-04-15

**Authors:** Anne-Lene Sand-Svartrud, Gunnhild Berdal, Maryam Azimi, Ingvild Bø, Turid Nygaard Dager, Siv Grødal Eppeland, Guro Ohldieck Fredheim, Anne Sirnes Hagland, Åse Klokkeide, Anita Dyb Linge, Joseph Sexton, Kjetil Tennebø, Helene Lindtvedt Valaas, Kristin Mjøsund, Hanne Dagfinrud, Ingvild Kjeken

**Affiliations:** 1 National Advisory Unit on Rehabilitation in Rheumatology, Division of Rheumatology and Research, Diakonhjemmet Hospital, PO Box 23, Vinderen, N-0319 Oslo, Norway; 2 Patient Advisory Board, Division of Rheumatology and Research Diakonhjemmet Hospital, Oslo, Norway; 3 Hospital for Rheumatic Diseases Lillehammer, Margrethe Grundtvigs veg 6, N-2609 Lillehammer, Norway; 4 Sørlandet Hospital Arendal, PO Box 416, Lundsiden, N-4604 Kristiansand, Norway; 5 Vikersund Rehabilitation Centre, Haaviks vei 25, N-3370 Vikersund, Norway; 6 Hospital for Rheumatic Diseases Haugesund, PO Box 2175, N-5504 Haugesund, Norway; 7 Rehabilitering Vest Rehabilitation Centre, PO Box 2175,, N-5504 Haugesund, Norway; 8 Muritunet Rehabilitation Centre, Grandedata 58, N-6210 Valldal, Norway; 9 Division of Rheumatology and Research, Diakonhjemmet Hospital, Oslo, Norway; 10 Valnesfjord Health Sports Centre, Østerkløftveien 249, N-8215 Valnesfjord, Norway; 11 Meråker Rehabilitation Centre, Østigardsveien 24, N-7530 Meråker, Norway

**Keywords:** Quality of health care, Quality indicators, Health services research, Rehabilitation, Musculoskeletal disease

## Abstract

**Background:**

The quality of provided health care may be an important source of variation in rehabilitation outcomes, increasing the interest in associations between quality indicators (QIs) and improved patient outcomes. Therefore, we examined the associations between the quality of rehabilitation processes and subsequent clinical outcomes among patients with rheumatic and musculoskeletal diseases (RMDs).

**Methods:**

In this multicentre prospective cohort study, adults with RMDs undergoing multidisciplinary rehabilitation at eight participating centres reported the quality of rehabilitation after 2 months and outcomes after 2, 7, and 12 months. We measured perceived quality of rehabilitation by 11 process indicators that cover the domains of initial assessments, patient participation and individual goal-setting, and individual follow-up and coordination across levels of health care. The patients responded “yes” or “no” to each indicator. Scores were calculated as pass rates (PRs) from 0 to 100% (best score). Clinical outcomes were goal attainment (Patient-Specific Functional Scale), physical function (30 s sit-to-stand test), and health-related quality of life (EuroQoL 5D-5L). Associations between patient-reported quality of care and each outcome measure at 7 months was analysed by linear mixed models.

**Results:**

A total of 293 patients were enrolled in this study (mean age 52 years, 76% female). Primary diagnoses were inflammatory rheumatic disease (64%), fibromyalgia syndrome (18%), unspecific neck, shoulder, or low back pain (8%), connective tissue disease (6%), and osteoarthritis (4%). The overall median PR for the process indicators was 73% (range 11–100%). The PR was lowest (median 40%) for individual follow-up and coordination across levels of care. The mixed model analyses showed that higher PRs for the process indicators were not associated with improved goal attainment or improved physical function or improved health-related quality of life.

**Conclusions:**

The quality of rehabilitation processes was not associated with important clinical outcomes. An implication of this is that measuring only the outcome dimension of quality may result in incomplete evaluation and monitoring of the quality of care, and we suggest using information from both the structure, process, and outcome dimensions to draw inferences about the quality, and plan future quality initiatives in the field of complex rehabilitation.

**Trial registration:**

The study is part of the larger BRIDGE trial (ClinicalTrials.gov NCT03102814).

## Background

Rheumatic and musculoskeletal diseases (RMDs) are major contributors to the overall need for rehabilitation services worldwide [1]. In the last few decades, the globally estimated number of years lived with disability has increased substantially due to the ageing of populations, the effects of unhealthy lifestyles, and other epidemiological and demographic factors [1]. Furthermore, patients with RMDs do not always receive sufficient benefit from medical treatment strategies. Consequently, some patients experience long-term declines in physical, psychological, or social functioning and may need rehabilitation services one or several times in their lives [1–4].

Rehabilitation is frequently described as a patient-centred process, reflecting how patients and health professionals engage with each other and collaborate towards the best possible function for the patients in interaction with their environments [5, 6]. A general consensus has been reached on the key components of high-quality rehabilitation, such as agreement on goals that are important to the patient, organized multidisciplinary delivery of goal-directed action plans, and coordinated care across care levels and institutions over time [5, 6]. Yet, the current delivery of these quality norms is suboptimal and varies across providers and geographic regions [7–9].

Progress towards more optimal delivery of rehabilitation may be aided by quality indicators (QIs), as these measures are designed to compare actual patient care to norms or ideal criteria [10]. Several QI sets are based on the expected relationships between three dimensions of quality: structure, process, and outcomes [10–15]. Structure indicators relate to the organization of the health service, available resources, and procedures [16, 17]. Process indicators relate to the actual provision and reception of the health service (activities and tasks), whereas outcomes are states of health, functioning, or wellbeing that follow the provided care and processes [16, 17]. However, we need more knowledge about the associations between structure, process, and outcomes in clinical contexts [17, 18]. As the quality of provided care may be an important source of variation in clinical outcomes, interest is growing regarding associations between the fulfilment of process indicators and the likelihood of improved patient outcomes [18–20].

In the field of RMDs, the relationship between process and outcome is inconsistent [21–27], and there are few studies from the specific area of rehabilitation. Therefore, our aim was to examine the associations between level of quality of the rehabilitation processes and subsequent clinical outcomes among patients with RMDs. More specifically, we aimed to explore whether higher quality as measured by patients’ responses to process indicators from a QI set for rehabilitation [11] is associated with better patient-reported outcomes in terms of goal attainment, physical function, and health-related quality of life (HRQoL).

## Methods

### Study design

This study was part of a large multi-centre study, the BRIDGE trial, which aimed to improve the quality, continuity, and coordination of rehabilitation for patients with RMDs [28]. In the trial, the effects of a new rehabilitation programme on patients’ goal attainment, physical function, and HRQoL were evaluated at admission, discharge, and after 2, 7, and 12 months. For this purpose, the BRIDGE trial was designed as a stepped-wedge, cluster-randomized, controlled trial comparing an intervention group (adding the new BRIDGE programme to the traditional programmes) with a control group (the traditional programmes) at eight participating rehabilitation centres in secondary health care in Norway. In short, elements in the BRIDGE programme were motivational interviewing, structured goal-setting, use of a written rehabilitation plan, tailored follow-up including plans for self-management, and individualized digital feedback and tools that patients could use to monitor their own progress and cooperate with others after discharge [28, 29].

In the present study, we analysed the patient sample as one cohort regardless of group allocation. This approach was considered to be the most suitable design for our study because it provided a larger variety of responses to the process indicators, as reported by the participants in the BRIDGE trial.

### Study population and recruitment

Eligible patients were ≥ 18 years old and admitted to 2–4 weeks of multidisciplinary rehabilitation care (inpatient at 7 centres, outpatient at 1 centre) due to inflammatory rheumatic diseases, systemic connective tissue diseases, osteoarthritis, fibromyalgia syndrome or chronic widespread pain, osteoporosis, or unspecific neck, shoulder, or low back pain (persistent for > 3 months). Further inclusion criteria were the ability to read and understand questionnaires in Norwegian and access to a smartphone or equivalent device for digital data collection, including a personal electronic credential for secure identification online. Exclusion criteria were fracture(s), cognitive impairment, or severe psychiatric disorders. Health professionals at eight rehabilitation centres in different regions of Norway performed the eligibility screening and inclusion procedures.

All included patients received verbal and written information about the study and provided written informed consent. The study was approved by the Norwegian Regional Committee for Medical Research Ethics (REK South-East, 2017/665). Two patient research partners were members of the trial steering committee and involved in all stages of the trial.

### Measurements

#### Time points for data collection

Patients were included from August 2017 to August 2018 and followed for 1 year. They used an online solution for self-reported health care assessments at admission (T_1_) and discharge (T_2_) from the rehabilitation stay, and at home 2, 7, and 12 months after admission (T_3_, T_4_, and T_5_, respectively). The patients answered the QI questionnaire only at T_3_. This time point was chosen to capture the patient perspective of the rehabilitation process in fair proximity to the rehabilitation stay, as well as in proximity to the first month of the follow-up period.

The patients reported goal attainment, physical function, and HRQoL at all five time points. In the present study, we only used the reports of these outcomes on T_4_ to allow for sufficient time after discharge for patients to implement goal-directed self-management strategies and lifestyle changes in their daily lives.

#### Background variables

We collected patients’ background characteristics at T_1_, when the following variables were used as covariates: age, sex, body mass index (BMI = weight [kg]/height^2^ [m^2^]), civil status (living with partner [yes/no]), education level (yes ≥ tertiary education), paid employment (yes = part- or full-time), comorbidities (yes ≥1 additional diagnosis), weekly physical training (yes = physical activities leading to increased heart rate and breathing for ≥30 min, minimum once a week), and smoking (yes = now and then, or more often).

#### Quality indicators

Supported by the Norwegian Health Directorate, a QI set for use in rehabilitation for RMDs has been developed by an expert panel comprising clinicians, researchers, and patient research partners [11]. This expert panel used a RAND/UCLA Appropriateness Method to reach consensus regarding evidence-based quality statements for quality in rehabilitation. Three dimensions of quality (structure, process, and outcome) were operationalized into 19 structure, 11 process, and 3 outcome QIs [11]. The set consists of two separate questionnaires; leaders at each centre respond to the first questionnaire, comprising the structure indicators, and patients respond to the other questionnaire, comprising the process and outcome indicators. As the content of several structure indicators matches the content of the process and/or outcome indicators, the set allows for measuring quality from the perspective of both the provider and the patient [11]. The QI set has been proven feasible, with satisfactory face and content validity, and adequate responsiveness in primary and secondary health care [11, 30].

In Table 1, we describe the 11 process indicators examined in this study. Patients answered yes or no to whether they had received the content addressed by each indicator. The indicators target a continuum of delivered care from several rehabilitation settings, most typically initiated in secondary care and followed up in primary care. Notably, the indicators target the overarching, interprofessional processes that aim to support the patient’s own rehabilitation process and increase the likelihood of desired outcomes. Consequently, the delivery of diagnosis- or profession-specific interventions is not directly measured by the process indicators. However, the indicators are expected to reflect the end product of general clinical reasoning and evidence-based interventions integrated throughout the rehabilitation process by health professionals*,* as experienced by the individual patient*.*

**Table 1:** Process indicators measuring quality in the rehabilitation process from the patient’s perspective [11]

Main theme		Process indicator number	Question (yes/no)
A	Initial assessments	P01	Were your health condition and life situation assessed during the first days of your rehabilitation period? *If “yes”, P02 is eligible:*
		P02	Did the assessments include both a physical examination and questions about mental and social conditions, network and home situation, and - if relevant – your work situation?
B	Patient participation and individual goal-setting through the rehabilitation process	P03	Was a written plan for the rehabilitation period developed that comprised your rehabilitation goals, what you should practice, etc.?
			Were you actively involved…
		P04	… in setting specific goals for the rehabilitation period?
		P05	… in preparing the specific written plan for the rehabilitation period?
		P06	Did you participate in at least two meetings with the team (or a health professional representing the team) during which your goal(s) and goal attainment thus far were discussed?
C	Patient participation, individual follow-up, and coordination across levels of health care		Were you asked if you wanted attendance in any of the meetings for…
		P07	… your next of kin?
		P08	… professionals you will relate to after the rehabilitation period?
		P09	Was a written follow-up plan developed for the period after rehabilitation, including what you were expected to work on yourself? *If “yes”, P10–11, are eligible:*
		P10	Did you participate in developing the follow-up plan?
		P11	As part of this plan, were you consulted about whether you needed follow-up from external professionals after the rehabilitation period?

We calculated the results as pass rates (PRs). The PR for a single indicator was “the total number of patients who answered yes for this particular indicator” divided by “the total number of patients who answered yes or no for the same indicator”. In addition, we calculated a summary PR score for each patient as the total of “yes” answers from the patient divided by the eligible QI items for the same patient. The minimum number of eligible items was 8 due to mandatory responses to P01 and P03–09 (Table 1). If the response was “yes” to P01, item P02 was also eligible. If the response was “yes” to P09, items P10–11 were also eligible. In conclusion, the number of eligible items was 11 if the patient answered “yes” to both P01 and P09, 10 if the response was “no” to P01, 9 if the response was “no” to P09, and 8 if the response was “no” to both P01 and P09 (Table 1). For both single indicator PRs and summary PRs, we normalized the scores to 100 to report the values as a percentage (0–100%, with 100% = best score).

For statistical analyses, we aimed to record the overall influence of the perceived quality of the rehabilitation process as reflected by the summary PR score. In addition, to distinguish between essential components of the rehabilitation process, we grouped the single indicators into three categories reflecting the main themes of the rehabilitation stay and follow-up-period as presented in Table 1: Group A, *Initial assessments* (P01 + P02); Group B, *Patient participation and individual goal-setting through the rehabilitation process* (P03-P06); and Group C, *Patient participation, individual follow-up, and coordination across levels of health care* (P07-P11). For each patient, we calculated a summary PR score for each group of indicators. The PR score for Group A was the total “yes” answers to P01 and P02 divided by the eligible QI items in Group A for that patient. The PR score for Group B was the total “yes” answers to P03 - P06 divided by 4, because eligible QI items in Group B is always 4. Finally, the PR score for Group C was the total “yes” answers to P07 – P11 divided by the eligible QI items in Group C for that patient. In the statistical analyses, we used the term “PR variables” for the summary PR scores and PRs for Group A, B, and C.

#### Clinical outcomes

The primary outcome in the BRIDGE trial was goal attainment after 7 months, as measured by the Patient-Specific Functional Scale (PSFS) [31, 32]. The PSFS has open-ended categories in which the patients report up to five important activites that they currently find difficult to perform due to their health condition. The experienced performance for each activity is scored on an 11-point scale (0–10), with 0 indicating “unable to perform” and 10 indicating “no problem at all “[32, 33].

The secondary outcomes were physical function and HRQoL. We used the 30-s sit-to-stand test (30secSTS) [33–35] to assess physical function. According to specific instructions, the patient, who is seated in a chair, rises to a full standing position and then sits down again. The patient completes as many full stands as possible within 30 s [33, 35]. HRQoL was measured by the EuroQoL 5D-5L (EQ5D-index and EQ5D-vas) [33, 36]. For the EQ5D-index, patients respond to five dimensions of health (mobility, self-care, usual activities, pain/discomfort, and anxiety/depression) from 1 (no problems) to 5 (extreme problems). In the EQ5D-vas, the patients rate their current health state on a 100-mm visual analogue scale, with 0 indicating “*The worst health you can imagine*” and 100 indicating “*The best health you can imagine*” [33, 36]. All of these instruments have been tested for psychometric properties with satisfactory results in Norwegian RMD populations [33].

We investigated the mean performance score for all reported goals for each patient, termed PSFS. In addition, we examined the first reported goal separately, termed PSFSA1, because the first goal set by the patient is reported to be the most reliable in terms of test-retest stability [32].

Furthermore, based on clinical experience and research [37], we knew that agreed rehabilitation goals for the follow-up period at home may differ from rehabilitation goals chosen for the rehabiliation stay. Therefore, patients and professionals in the BRIDGE trial were allowed to either agree on new PSFS goals at discharge or pursue the PSFS goals defined at admission. Consequently, though T_1_ was the time point for baseline values for the 30secSTS, EQ5D-index, and EQ5D-vas, T_2_ was the baseline time point for PSFS.

#### Rationale for the expected process-outcome relation

According to Donabedian’s model for evaluating the quality of care, a good structure should increase the likelihood of a good process, and a good process, in turn, should increase the likelihood of good outcomes [17]. High-quality rehabiliation, as operationalized in the process indicators, aims to address patient-specific goals, physical function, and HRQoL either directly or indirectly through provided interventions, guidance, communication, and coordination. The rationale for these process-outcome assumptions was an inherent part of the RAND/UCLA process used to develop the QI set for rehabilitation [11]. To build consensus, the members of the panel rated proposed quality indicators according to the Organisation for Economic Co-operation and Development criteria in the Heath Care Quality Project [11, 38]. These criteria included considerations of the importance of what is being measured and how the health care system can take specific actions to improve their performance and ultimately, improve, maintain, or restore the patients’ health status and desired outcomes [11, 38]. To be approved, each indicator needed a sufficient foundation in terms of available, scientific evidence or evidence from the opinions of the broad expert panel [11, 38]. Thus, development of the QI set for rehabilitation was based on *quality of care*, which was defined by the Institute of Medicine [39] as “the degree to which health services for individuals and populations increase the likelihood of desired health outcomes and are consistent with current professional knowledge,” and *rehabilitation* is understood as a planned and coordinated process that reaches across levels of care, is tailored to the patient’s experiences and goals, and assists the individuals in their own efforts to achieve their best possible functioning and coping [11, 40].

### Statistical analysis

In the statistical analyses, we included all participants from the BRIDGE trial who completed the QIs at T_3_. We analysed data in STATA IC, version 16, and set the statistical significance level to 0.05.

We performed descriptive analyses to report demographic data, quantify the quality of the received rehabilitation process, and describe the observed changes for each clinical outcome, calculated as the outcome score at T_4_ minus the score for the same outcome at baseline. For the actual process indicators, there was no former established PR cut-off for high-quality care. Therefore, we used quartiles (0–25% = Q1, 25.1–50% = Q2, 50.1–75% = Q3, 75.1–100% = Q4) for the quality variable when we examined changes in outcomes by summary PR score for the process indicators.

As a preparatory analysis, we performed two regressions treating the summary PR for the quality variable as the response variable. In the first analysis, we regressed R on study centre alone. In the second regression, PR was regressed on the baseline predictors (age, sex, BMI, weekly training, comorbidity, paid employment, education level, civil status, and smoking), in addition to study centre.

#### Main analysis

We used a linear mixed model approach to assess the association between the process dimension of the quality of rehabilitation (the PR variable) and the study outcomes (goal attainment, physical function, and HRQoL, respectively). First, our primary independent variable was the summary PR for the process variables. For each outcome, its value at T_4_ was treated as the response, and the fixed effects were its baseline value, the PR variable, and a variable capturing elapsed time since study start. To account for centre level clustering, we included centre as a random effect in the basic model. In the fully adjusted model, we included a wider range of baseline predictors: age, sex, BMI, weekly training, comorbidity, paid employment, education level, civil status, and smoking. In a separate analysis, the primary independent variable was replaced by the three summary PR values for the single indicators grouped into categories (Group A-C). We used the same basic and fully adjusted models as described above.

For each outcome, three models were fit: one without PR variable(s) (*null model)*, one with the summary PR (to examine the quality variable as a sum score; *alternative model I)*, and one with PRs for Groups A to C (to examine the quality variable as composed of the three PR variables; *alternative model II*). Subsequently, the association between the quality PR and the outcome was assessed by the likelihood ratio test, comparing each of the latter two models to the first. In other words, we used the likelihood ratio test to examine whether the *alternative model (I or II, respectively)* provided significant improvement (better fit) over the *null model*.

For the main outcome, we also performed mixed-logistic regression analyses in order to differentiate between those attaining minimal clinically important difference (MCID) for PSFS, and not. MCID for PSFS is 2 or more points [32] and therefore, we evaluated PSFS as a dichotomized outcome (change > = 2 yes/no). This was done first for PSFS, and next for PSFS-A1.

## Results

A total of 293 (78%) of the 374 BRIDGE trial participants completed the QI questionnaire at T_3_ and were included in the current analysis. The participants were mostly female (76%), with a mean age of 52 (±12.3) years. They were referred to multidisciplinary rehabilitation most frequently due to inflammatory rheumatic disease (64%) or fibromyalgia syndrome (18%). Fifty percent of the study cohort had other chronic diseases (≥ 1 comorbidity) in addition to their primary diagnosis. Median disease duration for the primary diagnosis was 17 years. All of the baseline characteristics are presented in Table 2.

**Table 2:** Baseline characteristics of patients in this cohort study

	Total (n = 293)
Age, years, mean (min, max)	52 (18, 81)
Gender, female, n (%)	224 (76)
Diagnosis, n (%)	
Inflammatory rheumatic disease (SpA, PsA, RA, JRA)	188 (64)
Osteoarthritis	12 (4)
Connective tissue disease (SLE, SS, PMR, MCTD)	17 (6)
Fibromyalgia syndrome, CWP	54 (18)
Unspecific neck-, shoulder- and low back pain (> 3 months)	22 (8)
Osteoporosis	0
Disease duration, years, median (min, max)	17 (0, 68)
Comorbidities, n (%)	145 (50)
Medication usage	
NSAIDs, n (%)	134 (46)
Disease modifying anti-rheumatic drugs (DMARDs), n (%)	55 (19)
TNF-inhibitors, Biosimilars, JAK-inhibitors n (%)	102 (35)
Analgesics, n (%)	194 (66)
Other drugs, n (%)	201 (69)
BMI, kg/m^2^, median (min, max)	28 (17, 66)
Smokers, n (%)	69 (24)
Snuff users, n (%)	21 (7)
Education > 12 years, n (%)	117 (40)
Paid work, n (%)	126 (43)
Recipients of social security benefits, n (%)	213 (73)
Living with partner, n (%)	201 (69)
Physical exercise ≥1 per week, n (%)	164 (56)
General activity ≥1 per week, n (%)	207 (71)

*SpA* spondyloarthritis, *PsA* psoriatic arthritis, *RA* rheumatoid arthritis, *JRA* juvenile rheumatoid arthritis, *SLE* systemic lupus erythematosus, *SS* Sjögren syndrome, *PMR* polymyalgia rheumatica, *MCTD* mixed connective tissue disease, *CWP* chronic widespread pain, Disease duration (symptom debut) and comorbidities are self-reported. *NSAIDs* nonsteroidal anti-inflammatory drugs, DMARDS include corticosteroids, *TNF* tumor necrosis factor, *JAK* Janus kinase, *BMI* body mass index (body weight/height^2^). Physical exercise: increased heart rate and breathing for 30 min or longer. General activity: social or cultural activities, hobbies, work

### Quality of the rehabilitation process

**Fig. 1:**
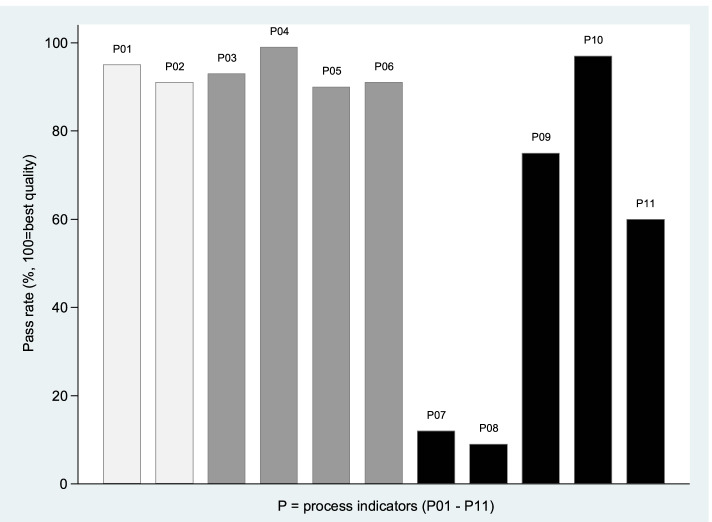
Patient-reported quality of rehabilitation. Pass rates for single process indicators (P01-P11), reported by 293 participants in the BRIDGE trial. *P01-P02 (light grey): initial assessments (group A). P03-P06 (grey): individual goal-setting through the rehabilitation process (group B). P07-P11 (dark grey): individual follow-up and coordination across levels of care (group C)*

The response rate for the patient-reported QI questionnaire was 100% (no missing items). The median summary PR score of the 11 process indicators was 73% (range 11–100%). For single indicators, the PRs ranged from 9 to 99%. We found the highest PR score for the indicator reflecting patient participation in tailored goal-setting (indicator P04), whereas the lowest PR scores were found for indicators regarding attendance in rehabilitation meetings for family or next of kin (indicator P07, PR score 12%), or important others/external professionals (indicator P08, PR score 9%; Fig. 1). When considering the single indicators grouped into the main themes, we found that the PR score was lowest (median 40%) for Group C, regarding individual follow-up and coordination across levels of care (Fig. 1).

### Patient-reported clinical outcomes

**Fig. 2:**
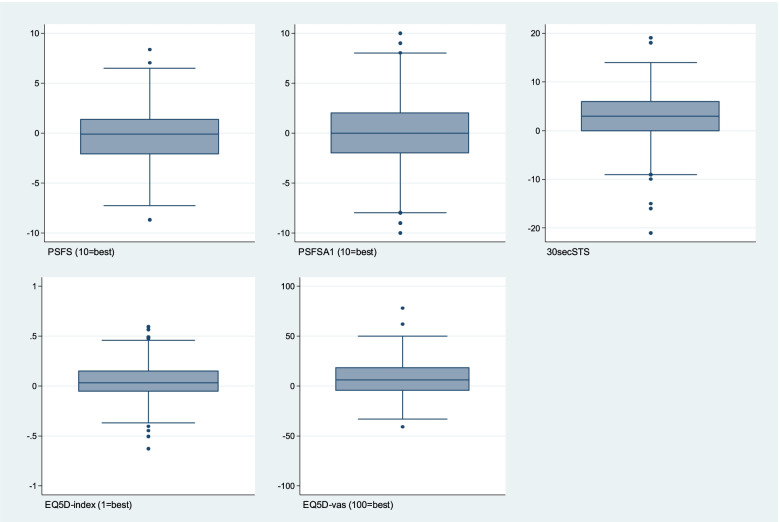
Distribution of change scores between 7 months and baseline for each clinical outcome in the BRIDGE trial. Positive values indicate improvements during the time period. *PSFS: Patient-Specific Functional Scale, mean performance score for all reported goals; PSFSA1: Patient-Specific Functional Scale, the performance score for the first reported goal only; 30secSTS: 30-s sit-to-stand test; EQ5D-index: EuroQoL 5D-5L index value; EQ5D-vas: EuroQoL 5D-5L visual analogue scale*

Available data and mean or median scores for the clinical outcomes at baseline and T_4_ (group level) are presented in Table 3. At the individual level, we found variation in the change scores for the period between baseline and T_4_ (Fig. 2). Though some individuals reported improvements, others experienced worsening or no change during the same period. This pattern was present for all clinical outcomes (Fig. 2).

**Table 3:** Patient-reported (*n* = 293) clinical rehabilitation outcomes at baseline and after 7 months (T_4_)

	Baseline		T_**4**_	
Outcome variable *(instrument, scale)*	No. of patients (%)	Value	No. of patients (%)	Value
**Goal attainment, all reported goals** *(PSFS, 0–10, 10 = best)*	291 (99%)	5.7 (SD 2.1)	228 (78%)	5.4 (SD 2.1)
**Goal attainment, first reported goal** *(PSFSA1, 0–10, 10 = best)*	288 (98%)	5.7 (SD 2.6)	226 (77%)	5.4 (SD 2.8)
**Physical function** *(30secSTS, higher number = better)*	285 (97%)	14.5 (SD 5.4)	235 (80%)	17.6 (SD 7.4)
**Health-related quality of life** *(EQ5D-index, [− 1,1], 1 = best)*	279 (95%)	0.66 (min 0.28, max 0.94)	231 (79%)	0.73 (min 0.11, max 1.00)
**Health-related quality of life** *(EQ5D-vas, 0–100, 100 = best)*	288 (98%)	47.2 (SD 17.7)	239 (82%)	54.6 (SD 19.4)

Values are presented as mean and standard deviation (SD) for normally distributed data, or median with the minimum and maximum. *PSFS* Patient-Specific Functional Scale, mean ability score for all reported goals, *PSFSA1* Patient-Specific Functional Scale, mean ability score for the first goal set by the patient, *30secSTS* 30-s sit-to-stand test, *EQ5D-index* EuroQoL 5D-5L index value, *EQ5D-vas* EuroQoL 5D-visual analogue scale

The preparatory analysis showed that around 90% of the variation in care quality was unexplained by centre (adjusted R-squared was 0.08) and case-mix (adjusted R-squared for the baseline predictors were 0.10).

### The process-outcome relation

**Fig. 3:**
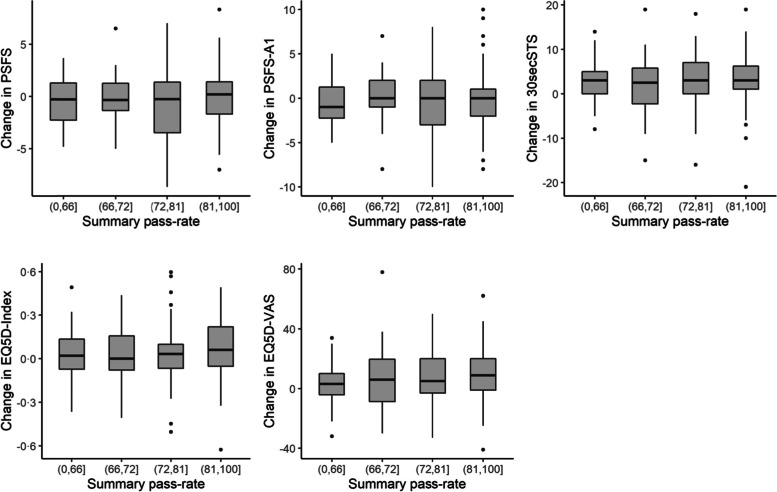
Change in outcome by summary pass rate quartile for the process indicators. A positive change score indicates improvements between baseline and 7 months. Pass rates in the highest quartile indicate highest fulfilment of the process indicators (best quality). *PSFSA1: Patient-Specific Functional Scale, the performance score for the first reported goal only; 30secSTS: 30-s sit-to-stand test; EQ5D-index: EuroQoL 5D-5L index value; EQ5D-vas: EuroQoL 5D-5L visual analogue scale*

As shown in Fig. 3, we found that changes in outcomes between T_4_ and baseline did not differ much when we examined these changes visually as PR scores for each quartile. Thus, from these initial descriptive analyses, we assumed that patients who reported higher fulfilment of the specified processes of rehabilitation (higher PRs) had only slightly or no better outcomes than patients who received less of the content addressed by the QIs (Fig. 3). The apparent lack of relationship between the quality of the rehabilitation process and the subsequent clinical outcome was confirmed in the mixed model analyses.

Results from the mixed model analyses showed that higher summary PRs for the process indicators were not associated with improved goal attainment, physical function, or HRQoL. As shown in Table 4, part 1 (quality variable as a sum score), the beta-coefficients ranged from 0.001 to 0.106 in the basic model and − 0.010 to 0.099 in the fully adjusted model. We found similar results when we examined the quality variable composed of the three PR variables for Group A, Group B, and Group C. None of the PR variables for the main themes in the rehabilitation process could explain the variance in any of the clinical outcomes (Table 4).

**Table 4:** Associations between quality indicators and clinical outcomes in mixed model analyses

**1. To examine the quality variable as a sum score*(clinical outcome (one by one) as the dependent variable)***						
			**Basic adjustments**		**Large adjustments**	
**Clinical outcome (instrument)**	**Quality variable**		**β (95% CI)**	***p*-value**	**β (95% CI)**	***p*-value**
Goal attainment, all reported goals (PSFS)	QI Summary PR score		0.010 (−0.009–0.030)	0.29	0.008 (− 0.013–0.028)	0.46
Goal attainment, first goal only (PSFSA1)	QI Summary PR score		0.015 (− 0.011–0.040)	0.26	0.017 (− 0.011–0.044)	0.23
Physical function (30secSTS)	QI Summary PR score		0.012 (− 0.039–0.063)	0.65	−0.010 (− 0.063–0.043)	0.71
Health-related quality of life (EQ5D-index)	QI Summary PR score		0.001 (− 0.001–0.002)	0.36	0.001 (− 0.001–0.002)	0.50
Health-related quality of life (EQ5D-vas)	QI Summary PR score		0.106 (− 0.044–0.255)	0.17	0.099 (− 0.060–0.258)	0.22
**2. To examine the quality variable as composed of three scores*(clinical outcome (one by one) as the dependent variable)***						
			**Basic adjustments**		**Large adjustments**	
**Clinical outcome (instrument)**	**Quality variables**		**β (95% CI)**	***p*-value**	**β (95% CI)**	***p*-value**
Goal attainment, all reported goals (PSFS)	QIs grouped into three main themes	PR score Group A	0.006 (−0.005–0.017)	0.26	0.003 (− 0.008–0.014)	0.62
		PR score Group B	− 0.001 (− 0.020–0.017)	0.90	−0.001 (− 0.021–0.018)	0.90
		PR score Group C	0.002 (− 0.008–0.012)	0.71	0.002 (− 0.009–0.013)	0.71
Goal attainment, first goal only (PSFSA1)	QIs grouped into three main themes	PR score Group A	0.004 (− 0.010–0.019)	0.56	0.001 (− 0.014–0.015)	0.94
		PR score Group B	0.009 (− 0.016–0.033)	0.49	0.016 (− 0.010–0.042)	0.23
		PR score Group C	−0.001 (− 0.015–0.013)	0.85	− 0.002 (− 0.016–0.013)	0.81
Physical function (30secSTS)	QIs grouped into three main themes	PR score Group A	0.022 (− 0.007–0.050)	0.14	0.014 (− 0.014–0.043)	0.32
		PR score Group B	− 0.011 (− 0.059–0.037)	0.66	−0.029 (− 0.079–0.022)	0.27
		PR score Group C	0.007 (− 0.020–0.034)	0.60	0.008 (− 0.020–0.035)	0.57
Health-related quality of life (EQ5D-index)	QIs grouped into three main themes	PR score Group A	0.000 (− 0.001–0.001)	0.90	0.000 (− 0.001–0.001)	0.86
		PR score Group B	0.000 (− 0.001–0.002)	0.78	0.000 (− 0.002–0.002)	0.92
		PR score Group C	0.000 (− 0.000–0.001)	0.29	0.000 (−0.000–0.001)	0.24
Health-related quality of life (EQ5D-vas)	QIs grouped into three main themes	PR score Group A	0.049 (−0.037–0.134)	0.26	0.034 (− 0.052–0.121)	0.44
		PR score Group B	− 0.007 (− 0.151–0.137)	0.92	−0.032 (− 0.188–0.124)	0.69
		PR score Group C	0.047 (− 0.031–0.125)	0.23	0.056 (− 0.025–0.137)	0.17

*β* beta-koeffisient. *CI* confidence interval, *QI* quality indicator, *PR* pass rate. “Basic adjustments” included fixed effects of the quality variable, the outcome’s baseline value, time (elapsed time since study start), and random effects of centre (clustering). “Large adjustments” added age, sex, BMI, weekly training, smoking, comorbidity, paid employment, education level, and civil status. *Group A* Initial assessments, *Group B* Individual goal-setting through the rehabilitation process, *Group C* Individual follow-up and coordination across care levels, *PSFS* Patient-Specific Functional Scale, mean performance score for all reported goals, *PSFSA1* Patient-Specific Functional Scale, mean performance score for the first activity set by the patient, *30secSTS* 30-second sit-to-stand test, *EQ5D-index* EuroQoL 5D-5L index value, *EQ5D-vas* EuroQoL 5D-visual analogue scale

The likelihood ratio tests resulted in *p*-values > 0.05 at different levels of adjustments, indicating that a model including the quality variable did not provide a better fit for the data than the simpler model without the quality variable. Thus, no significant associations were found between the process PRs and any of the outcome variables. Analyses with the main outcome as a dichotomized variable gave the same results.

### Additional analyses

During the observation period of this study, the support from health professionals was intended to decrease as the degree of patients’ self-management increased. The choice of T_4_ was to allow sufficient time for patients to establish self-management strategies and new, goal-directed habits in daily life, but the long interval (7 months after admission) may have challenged the recommended proximity of the outcomes to the received process of care [18]. Especially in cases with only brief, if any, contact with health professionals during the follow-up period, it may be questionable to attribute differences in outcomes to the rehabilitation received months ago. Therefore, to attain better proximity to the provided rehabilitation care, we performed additional analyses by replacing outcome data at T_4_ with data collected at T_3_ (2 months after admission). However, we did not find any associations between the process PRs and any of the outcome variables.

## Discussion

In this study, we did not find any associations between the quality of provided rehabilitation processes and subsequent clinical outcomes of multidisciplinary rehabilitation for adults with RMDs. The PR values for the process indicators were not associated with improvements in either patient-specific goal attainment, physical function, or HRQoL measured 7 months after the initiated rehabilitation process in the BRIDGE trial.

Regarding PRs, we found lower values for QIs within the domain of follow-up and coordination compared to PR values for indicators regarding initial assessments and tailored goal-setting. These findings may indicate a suboptimal transition between the rehabilitation process introduced in secondary care and the expected continuation in a community-based setting. As highlighted by others [41], improved rehabilitation outcomes for people with RMDs are more likely to be realized if support is established over a longer period of time. It can be argued that some indicators, such as involvement of next of kin or important others in the community, may not be applicable to all individuals [18]. However, results from the BRIDGE pilot study highlight that 98% of the patients report a need for follow-up from primary health care or other services, most frequently from a general practitioner, a physiotherapist, or the Norwegian Labour and Welfare Service [42]. In the same pilot study, an association was found between planned and received follow-up care and adherence to self-management activities [42]. As shown in other studies and stated by different health authorities, coordination across services is important in a high-quality rehabilitation process but often among the weakest elements in the rehabilitation trajectory [8, 9, 11, 43–46]. Therefore, further efforts are needed to attain an extended rehabilitation process after discharge [8, 9, 11, 43–46]. Such efforts should target the process dimension of quality in terms of tasks performed in the patient-professional cooperation. Equally important are efforts towards the structure dimension of quality, such as referral routines and information flow between providers and affiliated services, and sufficient competence and human resources at all levels of care being allocated and used in the best possible manner to facilitate a seamless transition of care and desired health outcomes for the patients [8, 9, 11, 43–46].

In contrast to what we hypothesized, patients who reported receiving higher quality rehabilitation did not report better outcomes at T_4_ compared to patients who reported a lesser quality process. One reason may be found in the relationship between patients’ outcome expectations and their agreements with clinicians regarding appropriate goal-setting. As the mean RMD duration in our sample was more than 15 years, some participants may have been striving towards maintenance of function as opposed to expectations of clinical improvement. Therefore, future quality initiatives and research should address both maintenance and improvement as desired outcomes [38]. In addition, rehabilitation outcomes are likely to be affected by factors beyond the issues covered by the selected quality indicators. Thus, the benefits of good quality may be lost or reduced during the follow-up period [18, 47]. Such concurrent factors may be fluctuating symptoms related to the RMD or additional health problems related to comorbid conditions [48, 49]. Clinicians’ interpersonal skills during the rehabilitation process may also vary and reduce the potential benefit, such as lower degree of careful listening or communication that is not adapted to the patient’s culture, level of health literacy, or other background characteristics [15]. Furthermore, at the patient level, other non-medical determinants of health are important for outcomes, such as aspects of the patients’ personal health behaviour after discharge, degree of life stress, lack of social support, or social events or duties inducing altered priority-setting, less available time, and less attention towards the ongoing rehabilitation process [38]. In our study, patients’ additional health challenges or non-medical determinants arising after discharge may not have been addressed sufficiently due to suboptimal coordination across care levels and less support from health professionals in the follow-up period. However, some variance in outcomes is probably influenced by factors beyond the variance in process quality. Thus, outcomes for patients with RMDs can be difficult to relate directly to the delivered process of rehabilitation [18, 48, 49]. Nevertheless, efforts should be made to improve the quality of rehabilitation processes as an independent contribution to the desired clinical outcomes [38].

Taken together, weak associations between the process quality and outcomes do not necessarily devalue the importance of a high-quality rehabilitation process. Methodological challenges in identifying associations between the process and patient outcomes have been reported by others [15, 18, 47–50]. In particular, as recognized in the BRIDGE trial, such challenges tend to occur when the delivered processes are complex and include several steps, longer periods of time are necessary to establish the desired outcomes, or the performance assessed by the outcomes is influenced more by the degree of patient adherence and selected self-management strategies than the provided care [18, 48]. Despite these challenges, the value of process indicators has been proposed to be important drivers of quality improvement because the use of these indicators may lead to improved awareness about the recommendations for optimal rehabilitation management and guide the clinical performance on a day-to-day basis [47, 50–52]. Though outcome measures are less informative about a problem related to delivery of care, the process indicators convey information about which parts of the rehabilitation practice have potential for improvement [15, 47, 48, 51, 52]. Such information may stimulate a dialogue between leaders and clinicians about appropriate actions to improve practices and step up the local quality initiatives and adoption of best practice recommendations [48]. Finally, process indicators may increase transparency regarding clinical processes and reduce unwanted differences in providers’ performance [52]. More research on associations between providers’ performance and the outcomes of rehabilitation is warranted. Strengths and limitations.

The strengths of this study include a large study cohort, a statistical methodology accounting for the hierarchical data structure, and a > 76% response rate 7 months after baseline. Furthermore, we evaluated QIs and outcomes from the same perspective (the patient perspective). This study also has some limitations. First, a small difference in quality when looking at PRs for Group A and Group B may reduce the potential to explain variations in outcome(s) at 7 months. Second, confounders, such as *self-efficacy, readiness for change,* and *health literacy,* could have been added in the analysis to better address potential self-management-related factors influencing the outcome [15]. Third, though the recruitment of patients from rural and urban regions and different rehabilitation settings may strengthen the generalizability of the findings in a Norwegian context, our results may not be applicable for settings and rehabilitation trajectories that differ significantly from the Norwegian health care system. Another limitation is the limited scientific evidence regarding why increased delivery of high-quality rehabilitation will lead to improvements in goal attainment, physical functioning, and HRQoL. However, expert opinions were used to supplement the available scientific evidence regarding each QI in the systematic development process [11]. There may also be a potential recall bias caused by the time point for measuring the QIs. Two months after the start of rehabilitation (i.e., T_3_), patients may not accurately remember the process they underwent before discharge. Nevertheless, at this first time point at home, their memory was probably helped by re-scoring the clinical outcomes, by a mandatory follow-up phone call from the rehabilitation centre between discharge and T_3_, and, optimally, the beginning of the extended care from the community. Lastly, limitations due to the yes/no format of the QI questionnaire yield information restricted to confirmed/unconfirmed for the content addressed by each indicator. Consequently, we did not know patients’ opinions on whether the goals were appropriately ambitious, whether plans for self-management and follow-up were feasible in their context at home, or whether potential barriers were identified and planned for. In future research, we will address these questions.

### Implications

This study is a first step to exploring associations between rehabilitation processes and the subsequent clinical outcomes using the process indicators from a QI set for rehabilitation in patients with RMDs. Our results support the need to promote the process indicators as a useful tool to be aware of, recognize, and deliver all aspects of best rehabilitation practice. Using the process indicators, we revealed that the quality of rehabilitation is still suboptimal regarding coordination between care levels and sufficient support for the patients’ self-management strategies after discharge. However, in rehabilitation, it can be difficult to relate the outcomes directly to the quality of the delivered rehabilitation process due to the additional influences of environmental factors and non-medical events arising along the highly personalized rehabilitation process. However, we consider information about clinical outcomes to be valuable and meaningful in evaluating and monitoring rehabilitation quality, but preferably in combination with information about the process dimension of quality.

For clinicians, improving the quality of rehabilitation processes may be difficult if the present structural conditions are disadvantageous. Therefore, quality initiatives from policymakers and leaders need to address structural factors aimed at increasing the likelihood of good processes, such as sufficient competence and resources in all care levels, written procedures, and establishment of good structures for mutual information flow and efficient referral routines. This broader perspective, including all dimensions of quality, applies well in complex rehabilitation, in which the health system, providers, and patients are mutually accountable for the clinical outcomes.

Results from this work may inform decisions on expected standards of rehabilitation services, such as stakeholders’ efforts to identify and reduce unwarranted variance in quality. Moreover, providers’ and receivers’ input on how quality initiatives apply and work in different contexts, will be essential in future work for further developing of national plans and indicators for quality improvement in rehabilitation.

## Conclusions

To the best of our knowledge, this study is the first examination of associations between the quality of rehabilitation processes and clinical outcomes based on the process indicators from a QI set for use in rehabilitation for adults with RMDs. We conclude that the quality of rehabilitation processes is not associated with subsequent clinical outcomes. An implication of this is that measuring only one dimension of quality may result in incomplete evaluation and monitoring of the quality of care, and we suggest using information from both the structure, process, and outcome dimensions to draw inferences about the quality and plan future quality initiatives in the field of complex rehabilitation.

## Data Availability

The datasets used and analysed during the current study are available from the corresponding author upon reasonable request.

## Abbreviations

QI: Quality indicator
RMD: Rheumatic and musculoskeletal disease
PR: Pass rate
PSFS: Patient-specific Functional Scale
30secSTS: 30 s sit-to-stand test
EQ5D-5 L: EuroQoL 5D-5L health-related quality of life
HRQoL: Health-related quality of life
BMI: Body mass index\

## References

[1] Cieza A, Causey K, Kamenov K, Hanson SW, Chatterji S, Vos T (2020). Global estimates of the need for rehabilitation based on the global burden of disease study 2019: a systematic analysis for the global burden of disease study 2019. Lancet..

[2] Kolasinski SL, Neogi T, Hochberg MC, Oatis C, Guyatt G, Block J (2020). 2019 American College of Rheumatology/Arthritis Foundation guideline for the Management of Osteoarthritis of the hand, hip, and knee. Arthritis Rheum.

[3] Macfarlane GJ, Kronisch C, Dean LE, Atzeni F, Häuser W, Fluß E (2017). EULAR revised recommendations for the management of fibromyalgia. Ann Rheum Dis.

[4] Chou R, Deyo R, Friedly J, Skelly A, Hashimoto R, Weimer M (2017). Nonpharmacologic therapies for low Back pain: a systematic review for an American College of Physicians Clinical Practice Guideline. Ann Intern Med.

[5] European physical and rehabilitation medicine bodies Alliance (2018). White book on physical and rehabilitation medicine in Europe. White book on physical and rehabilitation medicine in Europe. Eur J Phys Rehabil Med.

[6] World Health Organization [and] The World Bank. World report on disability. Geneva: World Health Organization; 2011.

[7] Wade D (2015). Rehabilitation – a new approach. Overview and part one: the problems. Clin Rehabil.

[8] Rapport om HelseOmsorg21. Et kunnskapssystem for bedre helse. Nasjonal forsknings- og innovasjonsstrategi for helse og omsorg. [The HealthCare21 Strategy. Research and innovation in health and care]. Available only in Norwegian.

[9] Supervision 2015 (2015). Norwegian Board of Health Supervision.

[10] Stelfox HT, Straus SE (2013). Measuring quality of care: considering measurement frameworks and needs assessment to guide quality indicator development. J Clin Epidemiol.

[11] Johansen I, Klokkerud M, Anke A, Børke J-B, Glott T, Hauglie U (2019). A quality indicator set for use in rehabilitation team care of people with rheumatic and musculoskeletal diseases; development and pilot testing. BMC Health Serv Res.

[12] Mahmood SB, Lesuis NMD, van Tuyl LHDP, van Riel PMDP, Landewé RMDP (2015). Quality in rheumatoid arthritis care. Best Pract Res Clin Rheumatol.

[13] Oostendorp RAB, Elvers JWH, Trijffel EV (2019). We are missing more. An international measurable model of clinical reasoning using quality indicators and routinely collected data. J Man Manip Ther.

[14] Gratacós J, Luelmo J, Rodríguez J, Notario J, Marco TN, de la Cueva P (2018). Standards of care and quality indicators for multidisciplinary care models for psoriatic arthritis in Spain. Rheumatol Int.

[15] Jesus T, Hoenig H (2015). Postacute rehabilitation quality of care: toward a shared conceptual framework. Arch Phys Med Rehabil.

[16] Mainz J (2003). Defining and classifying clinical indicators for quality improvement. Int J Qual Health Care.

[17] Donabedian A (1988). The quality of care. How can it be assessed?. JAMA..

[18] Parast L, Doyle B, Damberg CL, Shetty K, Ganz DA, Wenger NS (2015). Challenges in assessing the process–outcome link in practice. J Gen Intern Med.

[19] Hemingway H, Croft P, Perel P, Hayden JA, Abrams K, Timmis A (2013). Prognosis research strategy (PROGRESS) 1: a framework for researching clinical outcomes. BMJ..

[20] Brady BL, Tkacz J, Meyer R, Bolge SC, Ruetsch C (2017). Assessment of rheumatoid arthritis quality process measures and associated costs. Popul Health Manage.

[21] Spitaels D, Vankrunkelsven P, Grypdonck L, Dusar FR, Aertgeerts B, Luyten FP (2020). Quality of care for knee osteoarthritis in primary care: a patient's perspective. Arthritis Care Res.

[22] Kernder A, Richter JG, Fischer-Betz R, Winkler-Rohlfing B, Brinks R, Schneider M (2020). Quality of care predicts outcome in systemic lupus erythematosus: a cross-sectional analysis of a German long-term study (LuLa cohort). Lupus..

[23] Dziedzic KS, Healey EL, Porcheret M, Afolabi EK, Lewis M, Morden A (2018). Implementing core NICE guidelines for osteoarthritis in primary care with a model consultation (MOSAICS): a cluster randomised controlled trial. Osteoarthr Cartil.

[24] Yazdany J, Trupin L, Schmajuk G, Katz PP, Yelin EH (2014). Quality of care in systemic lupus erythematosus: the association between process and outcome measures in the lupus outcomes study. BMJ Qual Saf.

[25] Dua AB, Aggarwal R, Mikolaitis RA, Sequeira W, Block JA, Jolly M (2012). Rheumatologists' quality of care for lupus: comparison study between a university and county hospital. Arthritis Care Res.

[26] Jansen MJ, Hendriks EJ, Oostendorp RAB, Dekker J, De Bie RA (2010). Quality indicators indicate good adherence to the clinical practice guideline on “OA of the hip and knee” and few prognostic factors influence outcome indicators: a prospective cohort study. Eur J Phys Rehabil Med.

[27] Harris JG, Maletta KI, Kuhn EM, Olson JC (2017). Evaluation of quality indicators and disease damage in childhood-onset systemic lupus erythematosus patients. Clin Rheumatol.

[28] National Library of Medicine (US); 2017 [last updated March 2020]. The BRIDGE Rehabilitation Trial (BRIDGE). Available at: The BRIDGE Rehabilitation Trial - Full Text View - ClinicalTrials.gov.

[29] Berdal G, Sand-Svartrud AL, Azimi M, Bø I, Dager TN, Eppeland SG, Fredheim GO, Hagland AS, Klokkeide Å, Linge AD, Sexton J, Tennebø K, Valaas HL, Aasvold AM, Kjeken I (2021). Bridging gaps across levels of care in rehabilitation of patients with rheumatic- and musculoskeletal diseases: a stepped-wedge cluster randomized trial.

[30] Sand-Svartrud A-L, Berdal G, Azimi M, Bø I, Dager TN, Eppeland SG (2021). A quality indicator set for rehabilitation services for people with rheumatic and musculoskeletal diseases demonstrates adequate responsiveness in a pre-post evaluation. BMC Health Serv Res.

[31] Stratford P, Gill C, Westaway M, Binkley J (1995). Assessing disability and change on individual patients: a report of a patient specific measure. Physiother Can.

[32] Moseng T, Tveter A, Holm I, Dagfinrud H. Pasient-Spesifikk Funksjons Skala: Et nyttig verktøy for fysioterapeuter i primærhelsetjenesten. [The Patient-Specific Functional Scale – A useful tool for physiotherapist working in primary care]. Available only in Norwegian: Fysioterapeuten. 2013;80(2).

[33] Klokkerud M, Dagfinrud H, Uhlig T, Dager TN, Furunes KA, Klokkeide Å (2018). Developing and testing a consensus-based core set of outcome measures for rehabilitation in musculoskeletal diseases. Scand J Rheumatol.

[34] Csuka M, McCarty DJ (1985). Simple method for measurement of lower extremity muscle strength. Am J Med.

[35] Jones CJ, Rikli RE, Beam WC (1999). A 30-s chair-stand test as a measure of lower body strength in community-residing older adults. Res Q Exerc Sport.

[36] EuroQol_Research_Foundation. EQ-5D-5L User Guide 2019. 2019. Available from: https://euroqol.org/publications/user-guides/.

[37] Berdal G, Sand-Svartrud A-L, Bø I, Dager TN, Dingsør A, Eppeland SG (2018). Aiming for a healthier life: a qualitative content analysis of rehabilitation goals in patients with rheumatic diseases. Disabil Rehabil.

[38] Kelley E, Hurst J (2006). Health Care quality indicators project: conceptual framework paper.

[39] Institue of medicine (1990). Medicare: a strategy for quality assurance.

[40] Forskrift om habilitering og rehabilitering (2011). Forskrift om habilitering og rehabilitering, individuell plan og koordinator (FOR-2011-12-16-1256). Lovdata. [Norwegian legal regulations on habilitation and rehabilitation]. Available in Norwegian from: Forskrift om habilitering og rehabilitering, individuell plan og koordinator - Lovdata.

[41] Yun D, Choi J (2019). Person-centered rehabilitation care and outcomes: a systematic literature review. Int J Nurs Stud.

[42] Valaas HL, Klokkerud M, Hildeskår J, Hagland AS, Kjønli E, Mjøsund K, et al. Follow-up care and adherence to self-management activities in rehabilitation for patients with rheumatic and musculoskeletal diseases: results from a multicentre cohort study. Disabil Rehabil, 2021:1–10. 10.1080/09638288.2021.2008523. 10.1080/09638288.2021.2008523 34846264

[43] World Health Organization (2018). Continuity and coordination of care: a practice brief to support implementation of the WHO framework on integrated people-centred health services.

[44] World Health Organization. Framework on integrated, people-centred health services Report by the Secretariat. Sixty-ninth world health assembly. Provisional agenda item 16.1. A69/39. 2016.

[45] World Health Organisation. Handbook for national quality policiy and strategy: a practical approach for developing policy and strategy to improve quality of care. World Health Organization. 2018. https://apps.who.int/iris/handle/10665/272357. License: CC BY-NC-SA 3.0 IGO.

[46] Prp IS-2975. Evaluering av opptrappingsplan for habilitering og rehabilitering (2017-2019) [Evaluation of the «Habilitation and Rehabilitation Escalation Plan». The Norwegian Directorate of Health.] 2020. KPMG på oppdrag fra Helsedirektoratet.

[47] Bracewell N, Winchester DE. Accreditation in health care: does it make any difference to patient outcomes? BMJ Qual Saf. 2021. 10.1136/bmjqs-2020-012533. 10.1136/bmjqs-2020-012533 33542065

[48] Suter LG, Barber CE, Herrin J, Leong A, Losina E, Miller A (2016). American College of Rheumatology White Paper on performance outcome measures in rheumatology. Arthritis Care Res.

[49] Cooper M, Rouhi A, Barber CEH (2018). A systematic review of quality measures for inflammatory arthritis. J Rheumatol.

[50] Bilimoria KY (2015). Facilitating quality improvement: pushing the pendulum Back toward process measures. JAMA..

[51] Mant J (2001). Process versus outcome indicators in the assessment of quality in health care. Int J Qual Health Care.

[52] Lilford RJ, Brown CA, Nicholl J (2007). Use of process measures to monitor the quality of clinical practice. BMJ..

